# Carnitine Supplementation in Chronic Hemodialysis Patients—A Literature Review

**DOI:** 10.3390/jcm14145052

**Published:** 2025-07-16

**Authors:** Marina Kljajić, Lea Katalinić, Lovro Krajina, Anja Kovačić, Marta Kovačić, Nikolina Bašić-Jukić

**Affiliations:** 1 Department of Internal Medicine, University Hospital Centre Zagreb, 10000 Zagreb, Croatia; 2 School of Medicine, University of Zagreb, 10000 Zagreb, Croatia

**Keywords:** l-carnitine, hemodialysis, supplementation

## Abstract

**Background/Objectives:** Carnitine deficiency is common in hemodialysis patients and may contribute to anemia, inflammation, dyslipidemia, and muscle symptoms. This review explores the potential benefits of L-carnitine supplementation in this population. **Methods:** A thorough literature search of the PubMed database was conducted to identify clinical trials and studies assessing the effects of L-carnitine supplementation on adult hemodialysis patients. Key outcomes included the effects on inflammation, lipid profile, anemia, glycemic control, and muscle function. **Results:** Evidence suggests that L-carnitine may reduce inflammatory markers and improve lipid profiles by lowering triglycerides and increasing high-density lipoprotein (HDL). Several studies reported reduced erythropoietin need and improved hemoglobin levels. However, some studies did not find benefits of carnitine supplementation on the mentioned parameters. Results for muscle cramps, glycemic control, and cardiac function remain inconsistent. **Conclusions:** L-carnitine supplementation shows potential benefits in the management of hemodialysis complications. However, further well-designed trials are needed to confirm efficacy and optimize treatment protocols.

## 1. Introduction

Carnitine (β-hydroxy-γ-trimethylammonium butyrate) is a water-soluble quaternary amine with several vital metabolic functions, with the main one being the transport of long-chain fatty acids across the inner mitochondrial membrane, thus providing a substrate for β-oxidation and energy production [1]. Chronic kidney disease and dialysis can cause a variety of metabolic disorders, including disturbances of carnitine homeostasis [2,3]. Leading symptoms of carnitine deficiency include muscle weakness alternating with muscle cramps, fatigue, hypotension, and cardiac disorders such as arrhythmias, and abnormal laboratory findings related to serum lipids and chronic inflammation parameters (C-reactive protein) as well as insulin resistance [4]. As symptoms in patients with uremia or end-stage renal disease (ESRD) tend to overlap with those caused by hemodialysis itself, a challenge in the differentiation of which state is the primary cause is a frequent occurrence. In this paper, the effects of carnitine supplementation in patients undergoing hemodialysis will be explored. Specifically, the potential benefits of carnitine supplementation on improving anemia, chronic inflammation, and lipid profile in hemodialysis patients will be investigated. Additionally, the paper will review current research on the mechanisms through which carnitine may exert its effects and discuss the varying results from clinical trials. Ultimately, this work aims to provide a comprehensive overview of carnitine supplementation as a therapeutic approach in the management of hemodialysis patients, highlighting both its potential advantages and the challenges that remain in its widespread implementation.

## 2. Materials and Methods

A comprehensive literature review was conducted to evaluate the role of carnitine supplementation in patients undergoing hemodialysis. The primary source of data was the PubMed database, accessing articles published between January 1980 and May 2025. The search strategy included combinations of the following Medical Subject Headings (MeSH) terms and keywords: “carnitine”, “L-carnitine”, “hemodialysis”, “dialysis”, “end-stage renal disease”, “ESRD”, and “supplementation”. Inclusion criteria were original research articles, randomized controlled trials, and clinical trials that examined the clinical effects or pharmacokinetics of carnitine supplementation in hemodialysis patients. Exclusion criteria included case reports, letters to the editor, conference abstracts, studies involving pediatric patients, and studies involving peritoneal dialysis patients only. All articles were screened by title and abstract for relevance. Full texts of potentially eligible studies were then reviewed for final inclusion. Data extracted from the selected studies included study design, sample size, form and dosage of carnitine supplementation, duration of intervention, and reported outcomes (e.g., effects on inflammatory markers, anemia, lipid profile). The quality of evidence was appraised descriptively based on study design and methodological rigor, though no formal risk of bias assessment was performed. Study selection and data extraction were performed independently by two authors, with disagreements resolved by consensus or by consulting a third author. To aid in the creation of conceptual figures, we used OpenAI’s ChatGPT (OpenAI, https://chat.openai.com, accessed on 15 June 2025) and BioRender (https://www.biorender.com, accessed on 20 June 2025) to generate draft illustration layouts, which were subsequently edited by the authors for accuracy and clarity.

## 3. Discussion

### 3.1. Carnitine Metabolism

Prior to engaging in the metabolic pathway with carnitine, long-chain fatty acids must first be activated to form acyl-CoA in a reaction catalyzed by acyl-CoA synthetase on the outer mitochondrial membrane [5]. Thus formed acyl-CoA cannot enter the mitochondria as its inner membrane is impermeable to this compound [6]. Therefore, the entry of acyl-CoA into the mitochondrial matrix depends on the carnitine shuttle system [7]. In the mitochondrial matrix, carnitine controls the acetyl-CoA/CoA ratio by scavenging excess acyl residues via an enzyme called carnitine acyltransferase; it facilitates a balance between the acetylcarnitine/carnitine and acetyl-CoA/CoA pairs [8]. This results in decreased suppression of several mitochondrial enzymes essential for the breakdown of glucose and amino acids, and thereby reduces toxicity caused by the accumulation of acyl-CoA [8,9]. Carnitine is also an osmolyte involved in the regulation of cell volume, fluid balance, and tonicity of the extracellular space [10]. Additionally, it takes part in oxidative stress reduction by inhibiting reactive oxygen species-producing enzymes and upregulating antioxidant enzymes. It also serves as an anti-apoptotic agent [11].

On average, humans obtain 75% of carnitine from the diet, primarily from meat, fish, and dairy products [12,13]. Biologically active stereoisomer, L-carnitine, is absorbed from the lumen of the small intestine into the enterocytes, and the unabsorbed portion is degraded by the bacteria in the large intestine [14]. The remaining 25% of carnitine is synthesized endogenously in the liver, kidneys, and brain from lysine and methionine, two essential amino acids [15,16]. Tissues such as cardiac and skeletal muscles lack γ-butyrobetaine hydroxylase, one of the enzymes required for carnitine synthesis, which is why they obtain carnitine from the blood [17,18]. After intestinal absorption or synthesis in the liver and kidneys, L-carnitine is distributed in the extracellular fluid after travelling through the bloodstream unbound to plasma proteins [19].

**Figure 1:**
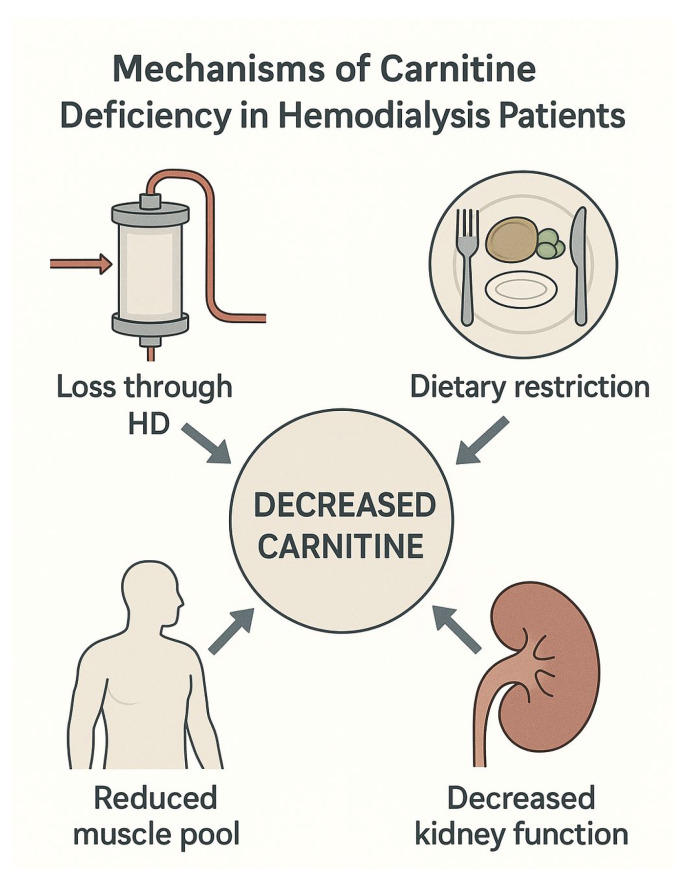
Illustration of mechanisms leading to decreased serum carnitine in hemodialysis patients.

As mentioned, chronic kidney disease and dialysis can cause a variety of metabolic disorders, including disturbances of carnitine homeostasis [2,3]. The de novo synthesis of carnitine in the kidneys and liver is limited by the availability of trimethyl-lysine, which is released during protein degradation. This process involves multiple enzymatic reactions that rely on essential nutrients such as vitamin C, iron, niacin, and vitamin B6 [20]. Hemodialysis (HD) patients are susceptible to water-soluble vitamin deficiencies. Causes include impaired absorption in the gastrointestinal tract, losses during HD, and inadequate dietary intake. The two-center cross-sectional study conducted in Poland showed that B1, B6, and B12 levels were notably lower in HD patients than in the control group [21]. They also measured vitamin B3 and C levels and found a positive correlation with albumin concentration, which suggested that individuals with better nutritional status tend to have higher levels of certain vitamins [21]. Vitamins play an important role as antioxidants, and their deficiency can impair carnitine synthesis. Carnitine exists as either non-esterified, referred to as free carnitine, or esterified, known as acylcarnitine. Healthy kidneys have a crucial role in maintaining homeostasis. Besides being involved in synthesis, they ensure tubular reabsorption, which exceeds 90% of the filtered load [19]. Hemodialysis removes more carnitine in the free than esterified form, resulting in decreased free carnitine in the serum (Figure 1). On the other hand, acylcarnitine is increased in both chronic kidney disease (CKD) and hemodialysis [22]. Skeletal muscles make up 98% of the body’s carnitine pool, which is sustained through the absorption of L-carnitine from dietary sources, endogenous biosynthesis, and efficient renal tubular reabsorption [19]. Since HD rapidly decreases plasma carnitine levels, it is important that it is followed by a gradual replenishment during the interdialytic phase, primarily sourced from pools such as skeletal muscles [23]. As HD patients are generally malnourished, they have decreased carnitine stores, which ultimately leads to carnitine deficiency (Figure 1). There is an interplay of multiple factors, including uremic anorexia, alterations in taste, increased catabolism caused by comorbidity and chronic inflammation, and loss of nutrients in dialysate, that make CKD patients prone to protein–energy malnutrition [24]. Patients lose 30–40 g of amino acids and peptides during one HD cycle, with 30–40% of the amino acids lost being essential. If a patient fails to consume adequate amounts of high biological value proteins and experiences significant protein loss through dialysis, the body begins to catabolize muscle tissue to meet its protein requirements. This process can progressively lead to muscle atrophy, fatigue, unintentional weight loss, and cognitive decline. Therefore, malnutrition, losses in dialysate, and kidney failure, as organs involved in the synthesis and reabsorption of carnitine, can all lead to carnitine deficiency in CKD patients.

A common factor in all the states mentioned above seems to be inappropriate processing of carnitine, leading to its reduced levels in the serum. When discussing carnitine deficiency associated with hemodialysis, we are talking about a special, almost one-of-a-kind condition that consists of elements related to both primary and secondary carnitine deficiency [25]. It is necessary to emphasize that while all the mentioned symptoms are a result of carnitine deficiency, they are also interconnected and can precipitate each other.

**Figure 2:**
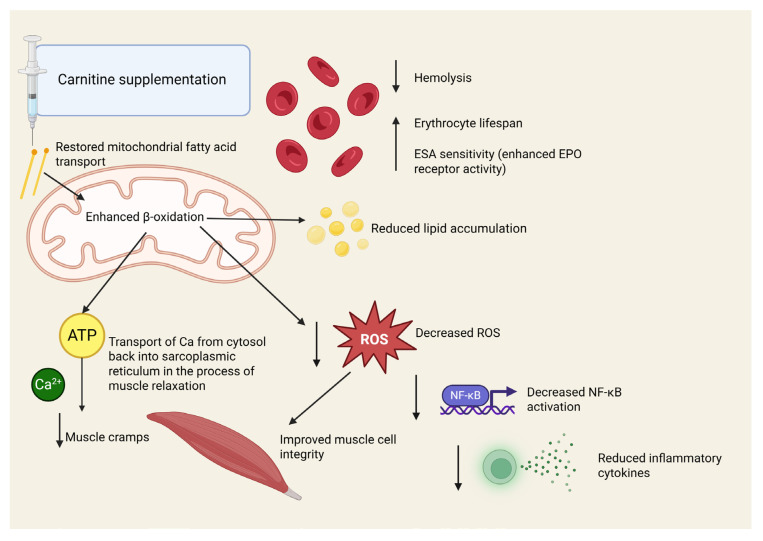
Proposed pathophysiological mechanisms of carnitine supplementation benefits in hemodialysis patients: effects on inflammation, anemia, lipid metabolism, and muscle cramps. Created in BioRender. Kljajić, M. (2025) https://BioRender.com/pmxrp3y (accessed on 20 June 2025). ROS—reactive oxygen species, Ca—calcium, ATP—adenosine triphosphate, ESA—erythropoietin-stimulating agents, EPO—erythropoietin.

Figure 2 outlines the proposed pathophysiological mechanisms underlying the clinical benefits of carnitine supplementation in hemodialysis patients. As mentioned, carnitine deficiency is common in this population due to reduced endogenous synthesis, impaired reabsorption, and losses during dialysis. Supplementation with L-carnitine may exert favorable effects through multiple interrelated mechanisms. It is thought to reduce systemic inflammation by modulating immune cell function, decreasing pro-inflammatory cytokines, and enhancing antioxidant defense [11]. It may inhibit reactive oxygen species (ROS) production and improve mitochondrial function, thereby attenuating the chronic low-grade inflammation commonly observed in hemodialysis patients. Furthermore, it supports erythrocyte membrane stability and energy metabolism, reducing red blood cell fragility and hemolysis. By facilitating the transport of long-chain fatty acids into mitochondria for β-oxidation, carnitine can improve lipid utilization and reduce triglyceride accumulation. Carnitine plays a key role in muscle energy metabolism, and its deficiency can lead to ATP depletion and impaired calcium reuptake in skeletal muscle, promoting sustained contraction and cramping. The mechanisms presented in this figure are further elaborated and supported by clinical evidence in the subsequent sections of the article.

### 3.2. Effect on Inflammatory Markers and Immune System

Low-grade inflammation occurs in all patients with chronic kidney disease, particularly hemodialysis patients, and contributes to increased mortality in this population [26,27]. Possible etiological factors include increased interleukin production stimulated by toxic uremic milieu, oxidative stress, acidosis, and decreased clearance of inflammatory cytokines by the kidneys [28,29,30]. In addition, complications related to vascular access, such as catheter sepsis and thrombosis of arteriovenous fistulae, favor inflammation [28,31,32]. The interaction between blood and dialysis membranes can trigger an inflammatory reaction through the activation of immune cells and complement systems [33,34]. Additionally, the water used in dialysis may contain traces of endotoxins despite strict purification measures [35,36]. Based on our literature search, there are seven available clinical trials that included control groups that investigated the effects of carnitine supplementation on inflammatory markers in hemodialysis patients (Table 1). The most frequently investigated inflammatory marker was C-reactive protein (CRP). The method of administration of L-carnitine was oral or intravenous. Dosages of orally administered L-carnitine varied between 500 and 1000 mg/day, while IV dosages varied between 10 mg/kg and 1 g/dose after each HD session. In the study by Duranay. M., et al. [37], repeated measures ANOVA was used to analyze the serial changes in clinical parameters between two groups, one with 21 patients receiving carnitine supplementation and the other with 21 patients not receiving supplementation, which served as a control. They found a statistically significant decrease in CRP levels in the carnitine group (*p* = 0.007), while CRP was significantly increased in the control group (*p* = 0.001). Other parameters analyzed, including total protein, albumin, and transferrin levels, showed improvement in the supplementation group, while white blood cell counts remained comparable within the two groups. The research conducted by Thomas S et al. [38] investigated the effects of L-carnitine on phagocytic cells in hemodialysis patients by conducting several experiments. Blood samples were collected before the initiation of treatment and at the end of the study period to isolate polymorphonuclear neutrophils and monocytes. The cells collected before the initiation of the study were exposed to various concentrations of L-carnitine or a control solution in vitro, and their phagocytic and respiratory burst activities were assessed. The researchers used a chemiluminescence assay to measure oxidative metabolism, a superoxide anion measurement to evaluate superoxide production, and a bacterial killing assay to test bactericidal activity against Staphylococcus aureus. They also assessed cell viability via lactate dehydrogenase release and measured phagocytosis with radiolabeled bacteria. The release of lactate dehydrogenase was reduced in phagocytic cells incubated in vitro with L-carnitine, suggesting enhanced viability of these cells; however, similar effects could not be demonstrated for the cells exposed to L-carnitine in vivo. Moreover, the results did not demonstrate any impact of L-carnitine on phagocytic function and viability in phagocytic cells isolated after treatment with carnitine in vivo. However, it is important to note that the study used a small dose of L-carnitine supplementation in patients compared to other studies (Table 1), as well as a short period of observation of only 4 months. Interestingly, Tabibi H. et al. [39] conducted a randomized controlled trial to evaluate the effects of L-carnitine supplementation on serum amyloid A (SAA) and several vascular inflammation markers—including soluble intercellular adhesion molecules (sICAM-1 and sICAM-2), soluble vascular cell adhesion molecule-1 (sVCAM-1), sE-selectin, sP-selectin, and oxidized low-density lipoprotein (oxLDL)—in hemodialysis patients. The study found a statistically significant 32% decrease in serum SAA levels (*p* < 0.001) in the L-carnitine group compared to baseline. However, there were no statistically significant changes in the vascular inflammation markers when comparing the L-carnitine group to the placebo group. Another unblinded, randomized clinical trial by Shakeri et al. [40] investigated the anti-inflammatory effects of L-carnitine supplementation in 36 hemodialysis patients with elevated lipoprotein (a) (Lp (a)) levels. The study reported significant reductions in serum CRP and interleukin-6 (IL-6) levels in the L-carnitine group—29% (*p* < 0.05) and 61% (*p* < 0.001), respectively, compared to baseline values. In contrast, no significant changes in CRP or IL-6 levels were observed in the control group. Additionally, there were no significant differences between the L-carnitine and control groups in terms of changes in interleukin-1β (IL-1β) and tumor necrosis factor-α (TNF-α) levels. Among the reviewed studies, this trial and that of Tabibi et al. [39] administered the highest oral dose of L-carnitine supplementation, at 1000 mg/day. Anti-inflammatory effects of L-carnitine supplementation were also reported in a controlled study by Suchitra M et al. [41], which used intravenous L-carnitine supplementation at a dose of 1 g intravenously after each hemodialysis session for 6 months. A significant decrease in CRP levels was observed among 20 patients in the L-carnitine-supplemented group (n = 20) compared to the control group (*p* < 0.05). Another placebo-controlled study by Savica V et al. [42] investigated the effects of intravenous L-carnitine supplementation on inflammation and nutritional status in the largest cohort of maintenance hemodialysis patients. The study included 113 patients and demonstrated a significant reduction in serum CRP levels (*p* = 0.002) after 6 months of treatment with L-carnitine at a dose of 20 mg/kg, administered three times per week. Notably, the overall reduction in CRP was predominantly observed in patients with baseline CRP levels above 3.0 mg/dL. A study by Orasan R et al. found no significant effect of L-carnitine supplementation on CRP levels in hemodialysis patients [43]. Notably, the dose used was 500 mg/day, administered orally.

In recent years, there has been a paradigm shift toward the personalization of dialysis therapy, aiming to mitigate complications such as chronic inflammation, which is highly prevalent in hemodialysis patients. Advances in dialyzer membrane technology, such as the use of high-flux and medium cut-off membranes, and the adoption of online hemodiafiltration, have demonstrated potential in reducing pro-inflammatory cytokine burden by enhancing the clearance of middle and large molecular weight toxins [44]. Additionally, the replacement of acetate with citrate in the dialysis buffer has emerged as a biocompatible alternative associated with reduced complement activation and improved hemodynamic stability [45]. These innovations underline the importance of individualized dialysis prescriptions based on patient-specific risk profiles, inflammatory markers, and tolerance. Incorporating such strategies may enhance the therapeutic efficacy of adjunctive treatments such as L-carnitine supplementation by providing a more favorable inflammatory milieu. This approach aligns with the broader goal of precision medicine in nephrology [44].

### 3.3. Effects on Lipid Profile

Patients with chronic kidney disease face an elevated risk of developing dyslipidemia because of reduced activities of lipoprotein lipase and lecithin cholesterol acyltransferase, along with lower hepatic lipase levels [46]. Statins are the first-line therapy in the treatment of hyperlipidemia in chronic kidney disease patients; however, their efficacy in hemodialysis patients is questioned [47]. Lp (a) serves as an independent risk factor for cardiovascular disease occurrence in individuals undergoing chronic hemodialysis treatment [48]. While multiple studies have investigated the effects of L-carnitine supplementation on the lipid profiles of hemodialysis patients, findings remain inconsistent.

Argani H et al. [49] investigated the effects of oral carnitine supplementation of 500 mg/kg over a period of 2 months in 40 HD patients and compared values prior to and after the treatment. They found that carnitine supplementation significantly lowered serum triglyceride and VLDL-C levels (Table 2). The results also showed a significant increase in high-density lipoprotein (HDL) in patients who received supplementation [49]. Furthermore, in the study by Naini AE et al., a significant improvement in total cholesterol, triglyceride, and LDL-cholesterol levels was observed in the intervention group, while no significant changes were observed in the control group [50]. They used a slightly higher dose of carnitine supplementation (750 mg/day orally) in the intervention group, during the same time as Argani H et al. [49]. In a placebo-controlled study by Guarnieri GF et al., eight patients receiving L-carnitine supplementation had significantly decreased levels of triglycerides after 14 weeks [51]. However, no changes were observed in serum cholesterol levels in either group. Notably, all selected patients had elevated baseline triglyceride concentrations (>200 mg/dL). Furthermore, no significant correlation was found between blood carnitine concentrations and serum triglyceride levels. Shojaei M et al. explored the effects of carnitine and coenzyme Q10 supplementation in 52 HD patients already receiving atorvastatin or lovastatin therapy [52]. They found a statistically significant lipoprotein (a) lowering effect (*p* = 0.01) in groups receiving carnitine and coenzyme Q10, as well as a combination of both, compared to placebo and baseline values, over a period of 3 months; however, no significant effects were observed on serum TC and lipoprotein levels. The dose of L-carnitine administered intravenously was 1000 mg 3× weekly (Table 2). The effects of carnitine supplementation on lipoprotein (a) levels were further explored by Shakeri et al. [40] (Table 1) in an unblinded, randomized controlled trial in 36 hemodialysis patients with lipoprotein (a) hyperlipoproteinemia. Oral L-carnitine supplementation at a dose of 1000 mg/day for 12 weeks had no statistically significant effect on lipoprotein (a) or oxidized LDL levels [40]. In a study of 20 hemodialysis patients with hypertriglyceridemia, Vacha GM et al. [53] evaluated the effects of L-carnitine (20 mg/kg IV post-dialysis) over 120 days, followed by a placebo phase in a crossover design. A significant reduction in triglyceride levels, along with increases in HDL cholesterol and apoprotein A, was observed predominantly in 12 patients characterized by elevated baseline triglycerides, low HDL cholesterol, and borderline-low apoprotein A levels. In a subset of non-responders, doubling the IV carnitine dose and extending treatment by 60 days subsequently resulted in a marked reduction in triglyceride levels [53]. Golper TA et al. [54] administered intravenous L-carnitine (20 mg/kg) to 38 hemodialysis patients after each dialysis session for up to six months. Their double-blind, placebo-controlled trial found no significant effects on lipid profiles [54]. Similarly, another study by Suchitra et al. (Table 1), which investigated the impact of intravenous L-carnitine (1000 mg three times per week post-dialysis) over six months, also failed to demonstrate any benefits on lipid profiles compared to the control group [41]. Interestingly, Weschel et al. [55] reported a paradoxical increase in plasma triglyceride levels following the initiation of oral L-carnitine supplementation at a dose of 3 g/day. Furthermore, due to a significant increase in platelet aggregation, they emphasized the thromboembolic potential of carnitine supplementation in high doses. In the study, they used 3000 mg/day of oral carnitine, which is the highest dose used among the listed studies; however, it is important to note that the study included only 10 patients, among whom six received L-carnitine supplementation during a 5-week trial [55]. Katalinić L et al. [56] found negative effects on the lipid profile in 50 hemodialysis patients receiving 1000 mg of L-carnitine intravenously at the end of every hemodialysis session for 12 months. Serum HDL cholesterol levels significantly decreased (*p* = 0.001), total cholesterol remained unchanged, and a notable rise in LDL cholesterol was observed (*p* = 0.005) [56]. An intriguing study by Vacha GM et al. investigated the effects of intravenous L-carnitine administration and addition to dialysate fluid among 22 HD patients [57] (Table 2). The study was conducted in three phases involving 22 HD patients. In the baseline phase, participants received 2 g of intravenous L-carnitine at the end of each dialysis session for 12 months. After a four-month washout period, patients were randomly assigned to two groups. In the therapy phase, both groups first received 1 g of intravenous L-carnitine post-dialysis for one month. This was followed by intradialytic administration of L-carnitine via the dialysate over three months—2 g per session for group 1 and 4 g per session for group 2. Both methods of L-carnitine supplementation (IV and via dialysate) helped reduce triglyceride levels, which had significantly increased during the washout period. Apoprotein A and HDL cholesterol levels also rose following initiation of IV therapy and addition of L-carnitine to the dialysate. While total cholesterol levels fluctuated during treatment, these changes were not statistically significant and remained within normal limits throughout the study [57]. Yderstraede KB et al. [58] supplemented the dialysis solution with carnitine to achieve a final concentration of 100 µmol/L in 21 hemodialysis patients over six months. However, no significant effects on lipid patterns were observed [58].

### 3.4. Effects on Anemia

Anemia associated with ESRD represents a significant problem in patients undergoing renal replacement therapy. Recombinant human erythropoietin (rhEPO) is a form of therapy that is often resorted to because of its ability to reverse renal anemia, even though, in order to maintain desired laboratory parameters associated with anemia, dosing is individual and varies in each patient [59,60,61]. Among other factors, in patients with ESRD, the presence of carnitine in serum and its quantity dictate the quality of response to rhEPO [62]. Studies of the effect of L-carnitine supplementation in maintenance hemodialysis patients have shown that this form of therapy has positive outcomes on red blood cell deformability, membrane stability, and increased hematocrit [59,63,64], leading to an assumption that endogenously synthesized carnitine plays a major role in the mentioned processes. In their prospective study, Kuwasawa-Iwasaki, M. et al. examined the effects of oral L-carnitine supplementation followed by IV supplementation over 24 months [65]. They found the overall increase in hemoglobin levels in 62 HD patients to be significant at both 12 and 24 months (Table 3) [65]. However, the study did not include a control group, which limits the ability to attribute observed effects solely to L-carnitine supplementation. A study from 1995 concluded that L-carnitine supplementation reduces erythropoietin dosage requirements in HD patients [59]. After treatment with L-carnitine, seven patients in the treatment group showed noticeable reductions in their need for recombinant human erythropoietin and were labelled as “responders”, while six patients showed no change and were considered “nonresponders.” Although responders had higher average erythropoietin needs and higher endogenous erythropoietin levels at the beginning of the study compared to nonresponders, these differences were not statistically significant. Kletzmayr et al. [66] categorized participants in their research into two groups: one group was administered intravenous L-carnitine (either 5 mg/kg or 25 mg/kg) following each dialysis session, while the other group was given a placebo. Both groups were administered intravenous iron saccharate (20 mg per HD session) during the initial four months, followed by a four-month period without iron supplementation [66]. They found L-carnitine supplementation to significantly decrease erythropoietin resistance index (ERI) in a group of patients receiving intravenous L-carnitine. Rathod R et al. discovered that administering intravenous carnitine at a dosage of 20 mg/kg following each HD session positively influenced hemoglobin levels in comparison to the placebo group [60]. Nonetheless, this was a single-blind study conducted over a brief period of 8 weeks (Table 3).

Several studies failed to prove the positive effects of carnitine supplementation on hemoglobin levels (Table 3). A study that included 40 HD patients found no statistically significant advantage of carnitine supplementation, administered orally at 500 mg/day, on hemoglobin and hematocrit levels after 2 months [49]. It is important to note that the study employed a pre–post design without a placebo or control group in a relatively short time period compared to other studies. Another study that failed to prove the benefits of carnitine supplementation on anemia was a double-blinded, placebo-controlled study that explored the effects of 1 g of IV carnitine supplementation after each HD session [67]. One year after continuous supplementation, there was no statistically significant increase in erythropoietin (EPO) responsiveness compared to the control group. Semeniuk J et al. [68] explored the supplementation effect on hemoglobin level as a secondary outcome in their placebo-controlled study on 16 HD patients. They used L-carnitine at a dose of 20 mg/kg for 12 months, followed by a 6-week washout period, and then crossed over to the alternate treatment for another 12 weeks. No difference in hemoglobin level was found regardless of the sequence of treatment [68]. However, this study’s statistical power was limited as it had only 16 participants. Furthermore, there was potential selection bias—inclusion criteria required patients to exhibit at least two specific symptoms (e.g., muscle cramping, hypotension), which may not represent the wider hemodialysis population, thereby limiting generalizability. An interesting study by Matsumura et al. investigated the relationship between serum carnitine levels and red blood cell fragility in 26 hemodialysis patients and postulated that one of the main causes of anemia in hemodialysis patients is hemolysis [69]. By measuring the levels of total, free, and acyl-carnitine in the blood, as well as the fragility of red blood cells using specific osmotic thresholds, they concluded that lower serum carnitine levels were significantly associated with greater red blood cell fragility. Moreover, patients with higher total and free carnitine levels required lower doses of EPO to maintain target hematocrit levels.

It is important to note that iron status, a key factor influencing erythropoiesis and EPO responsiveness, was not consistently reported across all reviewed studies; therefore, it is not possible to conclusively attribute the observed variability in hemoglobin response or EPO dose adjustment to L-carnitine supplementation alone. Future studies should incorporate standardized assessment of iron status to clarify the interaction between iron availability and the effects of carnitine on anemia.

### 3.5. Effects on Muscle Cramps/Weakness

The exact mechanism underlying muscle cramping in carnitine-deficient patients is not completely understood. As carnitine is one of the major components of β-oxidation, playing an important role in the translocation of long-chain fatty acids from the cytosol into the mitochondria [70], it is present in large quantities in skeletal muscle tissues [71]. Many studies have suggested a link between muscle weakness and abnormal carnitine levels in ERDS patients, leading Spagnoli et al. [72] to analyze the outcome of L-carnitine administration on human muscle fibers using morphometric parameters. L-carnitine was administered to patients undergoing hemodialysis, and over the course of the research, an increase in serum and muscle carnitine levels, as well as hypertrophy of muscle fibers, was observed; type 1 muscle fibers were more prevalent. Cellular levels of adenosine triphosphate, a molecule essential in the transport of calcium from the cytosol back into the sarcoplasmic reticulum during muscle relaxation, are reduced as a result of carnitine deficiency. The outcome of the mentioned mechanism is prolonged muscle contraction, which leads to muscle cramps. While nocturnal leg cramping is a fairly ordinary symptom reported in a great number of adults, carnitine deficiency, present in ERDS patients, is a potential cause of this condition. Patients on hemodialysis are at higher risk of experiencing muscle cramping because of a strict protein-restricted diet regimen, which consequently leads to reduced ingestion of carnitine, and loss of carnitine from the system during dialysis [3,73,74,75]. Using a questionnaire, Kuwasawa-Iwasaki M et al. [65] found that 58% of their patients experienced muscle cramping. No statistically significant improvement in muscle cramping was reported after administration of L-carnitine [65]. Conversely, in a randomized, double-blind, placebo-controlled study involving 82 dialysis patients, muscle cramps showed reduction solely in the group treated with carnitine [76]. The research conducted by Vaux et al. examined whether administering intravenous L-carnitine at a dosage of 20 mg/kg three times a week after dialysis could enhance muscle metabolism in individuals receiving hemodialysis [77]. During a 16-week timeframe, 26 patients were randomly assigned to receive either L-carnitine or a placebo. Muscle bioenergetics and functionality were evaluated through 31P magnetic resonance spectroscopy, 1H magnetic resonance imaging, and near-infrared spectroscopy both at baseline and post-intervention. No advantages regarding muscle function were observed.

### 3.6. Effects on Glycemic Control

L-carnitine plays a significant role in glycemic control through multiple pathways. By maintaining an optimal acetyl-CoA/CoA balance, it enhances Pyruvate Dehydrogenase Complex activity, which allows the conversion of pyruvate into acetyl-CoA and is a crucial step in glucose oxidation [78,79,80]. Another mechanism includes modulation of glycolytic and gluconeogenic enzymes; more specifically, it upregulates genes involved in glycolysis and downregulates genes involved in gluconeogenesis [80]. Some also suggest that carnitine modifies insulin signaling pathways, enhances insulin secretion, and has a direct glucose-lowering effect similar to insulin [80,81]. Finally, L-carnitine enhances insulin-like growth factor 1 signaling, which may contribute to better glycemic control [80]. Zamani et al. conducted a meta-analysis on the outcome of L-carnitine supplementation on laboratory markers of glycemia [82]. The study found that L-carnitine uptake improves glycemic markers through reduction of fasting blood glucose, glycosylated hemoglobin, and HOMA-IR (homeostatic model assessment for insulin resistance) with lesser effects on insulin levels in the serum. For these positive effects to be detected, L-carnitine uptake must last for about 50 weeks. Doses higher than 2 mg/day were shown to be more effective when considering the reduction of insulin levels in the serum. The effect of L-carnitine supplementation on glycemic control can additionally be improved if it is administered alongside a hypocaloric diet [83].

### 3.7. Effects on Cardiac Function

Cardiomyopathy can be present in patients with ERDS and can lead to failure of cardiac function in the mentioned population. While hemodialysis is a common form of therapy in these patients, it cannot resolve cardiac dysfunction, leading to the conclusion that circulating toxins that are removed from the system during hemodialysis are not a factor that causes these problems. Bohmer et al. found that carnitine concentrations were low in skeletal and cardiac muscles [84]. Apart from a possible direct impact on cardiac muscle function, carnitine is likely responsible for indirect impacts on the cardiovascular system. TNF-α, which is present in higher quantities in ERDS patients [85], blocks phosphorylation of insulin receptors, contributing to insulin resistance in these patients [86]. While some studies, such as that by Sakurabayashi et al. [87], show structural improvements in the heart with long-term oral L-carnitine supplementation, others report no functional improvement with short-term intravenous supplementation [88]. These mixed results highlight the complexity of the condition and suggest that further research is needed to clarify carnitine’s therapeutic potential in cardiac outcomes in ESRD patients.

### 3.8. Limitations

The lack of a formal risk of bias assessment is a limitation of this review, and the findings should be interpreted cautiously.

## 4. Conclusions

L-carnitine supplementation in hemodialysis patients shows promising potential in addressing several common complications associated with chronic kidney disease. Evidence suggests that it may contribute to the improvement of dyslipidemia by reducing triglyceride levels and potentially increasing HDL cholesterol. Additionally, L-carnitine has been associated with a reduction in erythropoietin requirements and improved hemoglobin levels, indicating a beneficial role in managing renal anemia. Furthermore, its anti-inflammatory effects, potentially through modulation of pro-inflammatory cytokines, highlight its role in mitigating the chronic inflammatory state prevalent in HD patients. While results vary depending on dosage, duration, and patient characteristics, L-carnitine appears to offer a supportive therapeutic option in the multifaceted management of patients undergoing maintenance hemodialysis. Further large-scale, long-term studies are warranted to better define optimal administration protocols and patient selection.

## Figures and Tables

**Table 1:** Studies examining the effects of carnitine supplementation on inflammatory parameters in hemodialysis patients.

Authors	Study Design	Number of Participants	Duration of Study	Dose of L-Carnitine	Route of Application	Conclusions
Effects on Inflammatory Markers						
**Duranay M et al.** **[****37****]**	Randomized NB controlled study	42	6 months	20 mg/kg 3× per week	IV	CRP decreased in the supplementation group
**Thomas S et al.** **[****38****]**	DB, randomized, PC study	17	4 months	10 mg/kg per dialysis session	IV	Failed to prove the benefits of carnitine supplementation
**Tabibi, Hadi et al.** **[****39****]**	randomized, DB, PC trial	36	12 weeks	1000 mg/day	oral	Reduced SAA compared to baseline values, no effect on vascular inflammation markers
**Shakeri A. et al.** **[****40****]**	Randomized NB controlled study	36	12 weeks	1000 mg/day	oral	CRP and IL-6 decreased; no effect on interleukin-1β, TNF-α; No effect on Lp (a) *
**Suchitra M et al.** **[****41****]**	SB, randomized, controlled study	35	6 months	1 g/dose 3× per week	IV	Decreased CRP in the supplementation group, no effect on lipid parameters *
**Savica V et al.** **[****42****]**	NR PC study	113	6 months	20 mg/kg 3× per week	IV	Decreased CRP, increase in serum albumin and transferrin, Hb level *
**Orasan R et al.** **[****43****]**	NR controlled study	31	3 months	500 mg/day	oral	No significant improvement in CRP, cholesterol, HDL, LDL, triglycerides, and hemoglobin levels* EPO dose

DB—double blind, NB—non-blinded, NR—nonrandomized, SB—single-blinded, PC—placebo-controlled, EPO—erythropoietin, Hb—hemoglobin, EPO—erythropoietin resistance index, IV—intravenous, kg—kilograms, mg—milligrams, CRP—C-reactive protein, IL-6—interleukin 6, Lp (a)—lipoprotein a, LDL—low-density lipoprotein, HDL—high-density lipoprotein, TNF-α—Tumor Necrosis Factor-alpha, SAA—serum amyloid A. Papers whose results are discussed in other sections and do not appear in other tables are marked with *.

**Table 2:** Studies examining the effects of carnitine supplementation on lipid patterns in hemodialysis patients.

Authors	Study Design	Number of Participants	Duration of Study	Dose of L-Carnitine	Route of Application	Findings
Effects on Lipid Profile						
**Argani H et al.** **[****49****]**	Prospective, single-arm interventional clinical trial	40	2 months	500 mg/day	oral	Significant decrease in serum TG and VLDL levels, increase in HDL levels No statistically significant change in Hb after the treatment *
**Naini AE et al.** **[****50****]**	Controlled clinical study	60	8 weeks	750 mg/day	oral	Significant decrease in TC, TG, and LDL in the supplementation group
**Guarnieri GF et al.** **[****51****]**	Randomized, PC clinical study	16	14 weeks	0.5 g for 8 weeks, 1.0 g for 6 weeks (both 3× per week)	IV	Decrease in serum triglyceride levels in the L-carnitine-treated group
**Shojaei M. et al.** **[****52****]**	Randomized, PC trial	52	3 months	1000 mg 3× per week	IV	Lp (a) reduced significantly in the carnitine, coenzyme Q10, and combination groups
**Vacha GM et al.** **[****53****]**	SB, crossover clinical study	29	120 + 120 days	20 mg/kg after each HD session	IV	Reduction in triglyceride levels, increase in HDL cholesterol and apoprotein A levels
**Golper TA et al.** **[****54****]**	DB, PC trial	82	6 months	20 mg/kg 3× per week	IV	No effect on lipid profile
**Weschler A et al.** **[****55****]**	Randomized, PC clinical trial	10	5 weeks	3 g/day	oral	Rise in triglyceride levels, rise in platelet aggregation
**Katalinic L et al.** **[****56****]**	Clinical controlled study	50	12 months	1 g after each HD session	IV	Significant increase in LDL and decrease in HDL. Triglyceride levels remained unchanged
**Vacha GM et al.** **[****57****]**	Clinical trial	22	Baseline phase: 12 months + washout: 4 months + 1 month + 3 months	2 g IV after every HD, 1 g IV after every HD. Carnitine in the dialysate fluid at concentrations of 2 g and 4 g	IV and in dialysate	Treatment with L-carnitine (intravenously and in the dialysate) decreased triglyceride levels and increased HDL and apoprotein (A) levels
**Yderstraede KB et al.** **[****58****]**	Controlled clinical study	21	6 months	Carnitine added to the dialysate to a final concentration of 100 µmol/L		No change in lipid pattern

DB—double blind, SB—single-blinded, PC—placebo-controlled, Hb—hemoglobin, IV—intravenous, kg—kilograms, mg—milligrams, g—grams, µmol—micromole, L—liters, Lp (a)—lipoprotein a, LDL—low-density lipoprotein, HDL—high-density lipoprotein, TG—triglycerides, TC—total cholesterol, HD—hemodialysis, VLDL—very-low-density lipoprotein. Papers whose results are discussed in other sections and do not appear in other tables are marked with *.

**Table 3:** Studies examining the effects of carnitine supplementation on anemia in hemodialysis patients.

Authors	Study Design	Number of Participants	Duration of the study	Dose of L-Carnitine	Route of Application	Findings
Effects on Anemia						
**Kletzmayr J et al.** **[****66****]**	DB, randomized, controlled trial	40	4 + 4 months	5 and 25 mg/kg after each HD session	IV	Decreased EPO RI
**Kuwasawa-Iwasaki M et al.** **[****65****]**	Prospective single-arm, non-randomized clinical study	62 HD	24 months	600 mg/day oral followed by 1000 mg three times/week	Oral + IV	Hb concentrations increased significantly, no improvement in muscle cramping
**Argani H et al.** **[****49****]**	Prospective, single-arm interventional clinical trial	40	2 months	500 mg/day	oral	No statistically significant change in Hb after the treatment
**Mercadal L et al.** **[****67****]**	Randomized, DB PC study	92	12 months	1 g after each dialysis session	IV	No improvement in response to EPO
**Semeniuk et al.** **[****68****]**	Randomized, PC, DB, crossover design	16	12 months–6 months washout period–12 months crossover	20 mg/kg	IV	No benefits on Hb level
**Labonia WD et al.** **[****59****]**	Randomized, DB, PC study	24	6 months	1 g after every HD session	IV	Reduction in EPO requirements
**Rathod R et al.** **[****60****]**	SB, randomized, PC clinical trial	20	8 weeks	20 mg/kg after every HD session	IV	Significant increase in Hb

DB—double blind, SB—single-blinded, PC—placebo-controlled, EPO—erythropoietin, Hb—hemoglobin, EPO RI—erythropoietin resistance index, IV—intravenous, kg—kilograms, mg—milligrams, g—grams, HD—hemodialysis.

## Data Availability

The raw data supporting the conclusions of this article will be made available by the authors upon request.
